# Attempts to replicate genetic associations with schizophrenia in a cohort from north India

**DOI:** 10.1038/s41537-017-0030-8

**Published:** 2017-08-30

**Authors:** Suman Prasad, Triptish Bhatia, Prachi Kukshal, Vishwajit L. Nimgaonkar, Smita N. Deshpande, B. K. Thelma

**Affiliations:** 1 Department of Genetics, University of Delhi South Campus, New Delhi, 110 021 India; 2 Department of Psychiatry, Center of Excellence in Mental Health, PGIMER- Dr Ram Manohar Lohia Hospital, New Delhi, 110 001 India; 3 Department of Psychiatry and Human Genetics, University of Pittsburgh School of Medicine and Graduate School of Public Health, Pittsburgh, PA 15213 USA

## Abstract

Schizophrenia is a chronic, severe, heritable disorder. Genome-wide association studies, conducted predominantly among Caucasians, have indicated > 100 risk alleles, with most significant SNPs on chromosome 6. There is growing interest as to whether these risk alleles are relevant in other ethnic groups as well. Neither an Indian genome-wide association studies nor a systematic replication of GWAS findings from other populations are reported. Thus, we analyzed 32 SNPs, including those associated in the Caucasian ancestry GWAS and other candidate gene studies, in a north Indian schizophrenia cohort (*n* = 1009 patients; *n* = 1029 controls) using a Sequenom mass array. Cognitive functioning was also assessed using the Hindi version of the Penn Computerized Neuropsychological Battery in a subset of the sample. *MICB* (rs6916394) a previously noted Caucasian candidate, was associated with schizophrenia at the *p* = 0.02 level. One SNP, rs2064430, *AHI1* (6q23.3, SZ Gene database SNP) was associated at the *p* = 0.04 level. Other candidates had even less significance with rs6932590, intergenic (*p* = 0.07); rs3130615, *MICB* (*p* = 0.08); rs6916921, *NFKBIL1* (*p* = 0.08*)* and rs9273012, *HLA-DQA1* (*p* = 0.06) and haplotypic associations (*p* = 0.01–0.05) of 6p SNPs were detected. Of note, nominally significant associations with cognitive variables were identified, after covarying for age and diagnostic status. SNPs with *p* < 0.01 were: rs3130375, with working memory (*p* = 0.007); rs377763, with sensorimotor (*p* = 0.004); rs6916921, *NFKBIL1* with emotion (*p* = 0.01). This relative lack of significant positive associations is likely influenced by the sample size and/or differences in the genetic architecture of schizophrenia across populations, encouraging population specific studies to identify shared and unique genetic risk factors for schizophrenia.

## Population genetics: Caucasians and Indians exhibit genetic disjunction in schizophrenia

A tenuous link between schizophrenia’s genetic basis in Caucasians and Indians calls for more comprehensive research on the latter. Large-scale analyses of the human genome have identified over a hundred genetic variations associated with schizophrenia; however, these have focused largely on European and North American populations. Researchers led by the University of Delhi’s BK Thelma, and Smita Deshpande of the Dr. Ram Manohar Lohia Hospital, India, selected 32 gene variations from past studies to look for similar associations in Indians. Many assays met limited success, though the team found significant correlations between certain variations and specific cognitive hallmarks of schizophrenia. Aside from differences in genetic architecture, the lack of adequate and comparable genetic data on schizophrenia in Indians may contribute to this apparent difference to schizophrenia in Caucasian patients. This shows a clear need for more schizophrenia genetic studies in India.

## Introduction

Evidence for a genetic etiology of schizophrenia (SZ) is available from family, twin and adoption studies, and the heritability has been estimated at ~70%.^1, 2^ However, it needs to be emphasized that these conclusions are based on data from Caucasian populations and little is known about whether they are similar in other racial groups. Overall, family based linkage analyses in all populations provided scant evidence for replicable risk factors.^3^ Though some genetic association studies relying on candidate neurotransmitters indicated suggestive associations, the associations were difficult to replicate.^4^ Encouragingly, Genome-wide association studies (GWASs) utilizing very large sets of samples and common variants densely covering the entire genome have yielded more consistent results.^5–7^ The most significantly associated SNPs were noted on chromosome 6p21–24 in an initial GWAS, while other groups reported associations with copy number variants and with cognitive endophenotypes.^8–12^ These too were largely performed in only Caucasian populations. More recently, Phase I and II of Schizophrenia Working Group of the psychiatric genomics consortium (PGC) reported significant associations at 108 loci for SZ in a sample of 21,000 cases and 38,000 controls^13^ of European descent; the strongest associations were also noted in the major histocompatibility (MHC) region on chromosome 6, known to be involved in immune function,^14^ consistent with earlier association studies.^15–17^ More refined analyses indicated associations with a copy number variation incorporating the C4A locus which was supported by post-mortem gene expression studies.^18^ Other associations, including genes related to Calcium channel functions and to histone methylation processes have been reported.^5, 19^ The chromosome 6p21–24 region is gene dense and harbors the HLA (human leukocyte antigen) or MHC (major histocompatibility) locus which includes more than 100 immune response genes and several other genes unrelated to immune function. On the other hand, the chromosome 6q region harbors the *AHI1* (human Abelson helper integration site-1) gene which has been shown to be associated with neurological, hematologic, and metabolic diseases.^20, 21^ Variation in MHC genes could be associated with SZ, in part through impaired immune responses, increase related to predisposition to viral infection(s),^22, 23^ neuroinflammation or even altered synaptic plasticity.^24^ Cognitive impairment, one of the deficits, central to SZ etiology^25^ has been observed consistently. A relatively high range of heritability (32–67%) for General Cognitive Ability was observed in a meta-analysis of > 800,000 subjects, likely due to differences in genetic and environmental effects, confirming that many cognitive phenotypes are under strong genetic influences.^26^ It is thought by many investigators that aspects of cognitive impairment may be endophenotypes or intermediate phenotypes that are more directly related to the genetic risk factors than the clinical illness of SZ itself. Almost all of these studies have been performed with samples of Caucasian ancestry.

Studies in other populations have been initiated, but have lagged in sample size compared with the Caucasian ancestry samples.^27^ Though the diverse Indian populations are genetically more closer to Caucasian populations^28–30^ they are notably different based on admixture analysis and poor portability of tag SNPs.^31, 32^ SZ is prevalent in India,^33^ but relatively few gene mapping studies have been conducted. The population is neither included in the large GWASs and PGC- GWAS, nor are there any studies using polygenic risk scores based on PGC data. In past two decades, a number of hypothesis-driven candidate gene variations, focused mainly on genes from the neurotransmitter and signaling pathways, were tested for their association with SZ, as well as tardive dyskinesia, cognition and response to antipsychotic drugs in the Indian population (Table 1). The sample size in these studies varied (see reviews^34, 35^). Of these, the most significant associations were observed for rs715505 (*p* = 0.00007) in *synaptogyrin-1* ^36^; and those with SNPs in *NRG1* were replicated^37, 38^ (Table 1). More recently, efforts to check for SNP frequencies across Asian populations have been made for SZ.^39^ A sub-group of these samples (*n* = 489) belong to South Asian (SAS) individuals comprising of Gujarati Indians, Pakistani Punjabis, Bangladeshi Bengalis, Sri Lankan Tamils, and Indian Telugus. Though the mean allele frequencies of the genetic variants did not significantly differ between these populations, the combination of these variants was suggested to increase the risk of SZ.^39^ Of note, rs13194504 near *C4A* locus from the strongly associated MHC region^18^ was found to be monomorphic in all the SAS populations (including north Indian population, our unpublished observation) and therefore was not analyzed further in our sample set. On the other hand, there has been only one study from India which tested association of PGC SNPs with SZ. This study using a custom panel of Illumina 1536 SNPs including PGC SNPs tested 351 patients and 385 controls from north India; 436 patients, 401 controls and 143 familial samples including complete and incomplete trio families from south India and identified a single PGC SNP rs548181 *STT3A*, (11q24.2) to be associated (*p* = 1.39 × 10^–^^7^, uncorrected; OR (95% CI) = 0.308 (0.181–0.524).^37^ In a different study of 35 miRSNPs with predicted functional relevance in 3′UTRs of genes shown previously to be associated with SZ, a significant association of miRSNP rs7430 in *PPP3CC* with SZ in a north Indian cohort has been reported.^40^ Further, an association (*p* = 0.001) of miRSNP rs9414688 *(CACNA1b)* and rs11136094 *(EGR3)* with cognitive domains of abstraction and mental flexibility and working memory respectively was also reported therein.^40^ Association of rs10497275 (*p* = 0.004) in *SCN1A*, in a subset of TD cases was also observed in the same study.^40^ Finally, using a whole exome sequencing strategy in multiplex SZ families from India, rare variants in *TAAR1* have been reported for the first time.^41^

**Table 1:** Previously reported candidate gene studies showing significant association with SZ cohorts from Indian population

(A) Markers associated with schizophrenia				
Gene	Marker	*p*-value	# of cases/controls	Reference
*IL1A*	rs1800587	0.03	488/186	Srinivas et al.^66^
*IL16*	rs1800795	0.04	492/488	
*TNFA*	rs361525	0.02	494/486	
*PPP3CC*	rs7430	0.001	1017/1073	John et al.^40^
*XRCC1*	rs25487	0.003	260/263	Sujitha et al.^67^
*GRM7*	rs3864075	0.004	840/876	Jajodia et al.^37^
*NRG1*	rs17603876	0.8 × 10^−^^4^	840/876	
*STT3A*	rs548181	0.1 × 10^−4^	840/876	
*DNMT1*	rs2114724	0.02	330/302	Saradalekshmi et al.^68^
*DNMT1*	rs2228611	0.002	330/302	
*SLC6A3*	rs403636	0.06	1007/1019	Kukshal et al.^69^
*DBH*	rs6271	0.004	1007/1019	
*SLC18A2*	rs363399	0.05	1007/1019	
*SLC18A2*	rs363338	0.07	1007/1019	
*SLC18A2*	rs10082463	0.07	1007/1019	
*SLC18A2*	rs363285	0.09	1007/1019	
*COMT*	rs933271	0.06	1007/1019	
*COMT*	rs4680	0.05	1007/1019	
*COMT*	rs9332377	0.02	1007/1019	
*NRG1*	rs35753505	0.04	1007/1009	Kukshal et al.^38^
*NRG1*	rs4733263	0.04	1007/1009	
*NRG1*	rs6994992	0.03	1007/1009	
*NRG1*	420M9-1355	0.02	1007/1009	
*NRG1*	rs1354336	0.01	1007/1009	
*NRG1*	rs10093107	0.008	1007/1009	
*NRG1*	rs3924999	0.02	1007/1009	
*NRG1*	rs11780123	0.01	1007/1009	
*COMT*	rs362204	0.028	215/215	Srivastava et al.^70^
*COMT*	rs4680	0.029	254/225	Gupta et al.^71^
*SLC6A4*	5HTTLPR	0.08	243/243	Vijayan et al.^72^
*SLC6A4*	rs20066713	0.001	243/243	
*SLC6A4*	STin2	0.001	243/243	
*DRD3*	rs10934254	0.03	141 trios	Talkowski et al.^73^
*SYNGR1*	rs909685	0.01	193/107	Verma et al.^36^
*SYNGR1*	rs715505	0.00007	193/107	
*SYNGR1*	rs6001566	0.03	193/107	
*NOTCH4*	197/198(GAAG)50	0.06	182 trios	Prasad et al.^74^
*NOTCH4*	(CTG)6	0.02	182 trios	
Ch 22 CAG repeat	22CH3	< 0.02	108/129	Saleem et al.^75^

(B) Markers associated with tardive dyskinesia and cognitive domains						
		TD*		Cognitive domain**		Reference
Gene	Marker	(n)	*p-*value	(*n*)	*p*-value	John et al.^40^
*SCN1A*	rs10497275	91/161	0.004	_	_	
*GCLM*	rs17881908	91/161	0.02	_	_	
*MTHFR*	rs4846049	91/161	0.02	152/292	Spatial ability = 0.01	
*PIP4K2A*	rs10734041	91/161	0.03	152/292	Face memory = 0.009; emotion = 0.03	
*CLDN5*	rs756654	91/161	0.04	152/292	_	
*MAOB*	rs2072745	_	_	152/292	Spatial ability = 0.02	
*NRG1*	rs17731664	_	_	152/292	Abstraction and mental flexibility = 0.04	
*COMT*	rs165728	_	_	152/292	Spatial memory = 0.05; emotion = 0.05	
*CACNA1b*	rs9414688	_	_	152/292	Emotion = 0.02; working memory = 0.002; abstraction and mental flexibility = 0.001	
*SCN1A*	rs1813502	_	_	152/292	Spatial ability = 0.03	
*DISC1*	rs16856322	_	_	152/292	Sensory motor and emotional processing = 0.03; attention = 0.03	
*CHGB*	rs2821	_	_	152/292	Working memory = 0.003	
*IL1RN*	rs9005	_	_	152/292	Spatial memory = 0.03; attention = 0.003; abstraction and mental flexibility = 0.05	
*EGR3*	rs11136094	_	_	152/292	Emotion = 0.03; working memory = 0.001	
*SYN2*	rs17036056	_	_	152/292	Emotion = 0.005	
*GCLM*	rs178819088	91/161	0.02	_	_	
*NRG1*	rs35753505	_	_	116/170	Emotion = 0.0331	Kukshal et al.^38^
*NRG2*	rs6994992	_	_	116/171	Attention = 0.047	
*SLC18A2*	rs363285	_	_		TMT b task = 0.025	

*n = positives/negatives for TD

**n = cases/controls for cognition

From the above description it is clear that though there have been moderate data from candidate gene based association testing with SZ among Indian population, neither an Indian GWAS nor a study attempting replication of the Caucasian SZ GWAS findings are available to date. The following report is our first attempt to examine selected SNPs from the previous Caucasian SZ GWAS findings (*p* < 10^−8^) or SNPs from other large-scale association studies or cataloged in a SZ gene database^42^ (*p* < 0.05) in a moderate sized SZ cohort from north India. We also tested for the first time their association with cognition in a subset of the same study cohort.

## Results

Of the 2038 samples, 67 individuals with a failure rate of ~20% for SNP assays were removed from the analysis. The demographic details of the remaining 1971 individuals are provided in Table 2. rs245201 from *CTXN3-SLC12A2* was not in Hardy–Weinberg equilibrium (*p* = 0.00004) and rs7597593 from *ZNF804A* had <95% calls; therefore these SNPs were removed from the analysis. Thus, a total of 1971 individuals and 30 SNPs (26 from chromosome 6 and four from other chromosomes, (Table 3) were taken forward for disease association.

**Table 2:** Demographic details of schizophrenia study cohort

(a) Distribution of age and gender in the study cohort				
Total samples	Cases (males/females)	Age in years mean ± SD	Controls (males/females)	Age in years mean ± SD
1971	980 (554/426)	30 ± 8.99	989* (554/433)	43 ± 13.75
(b) Distribution of samples with cognition data				
Cases (*n* = 189)			Controls (*n* = 119)	

*No gender information for two individuals

**Table 3:** SNPs nominally associated with schizophrenia (this study)

Sl no	CHR	Gene	SNP ID	Physical position	MA	MAF CA	MAF CO	Allelic p(df = 1)	OR (95% CI)	Genotypic p (df = 2)	Source for SNP selection^#^
1	2	*ZNF804A*	rs7597593*	184668853	C	0.5	0.47	–	–		Riley et al.^60^
2	2	*ZNF804A*	rs1344706	184913701	G	0.4	0.35	0.43	1.05 (0.92–1.20)	0.65	O’Donovan et al.^57^
3	5	*CTXN3-SLC12A2*	rs245201*	127833520	G	0.5	0.44	–	–		Potkin et al.^58^
4	6	*BTN3A2*	rs9393709**	26473126	T	0.3	0.35	0.64	0.97 (0.85–1.11)	0.54	Bamne et al.^23^
5	6	*Intergenic*	rs926300	27167422	A	0.1	0.09	0.86	1.02 (0.82–1.27)	0.98	Shi et al.^16^
6	6	*Intergenic*	rs13219181	27244204	G	0.1	0.1	0.67	1.04 (0.85–1.29)	0.9	Glessner et al.^12^
7	6	*Intergenic*	rs13194053	27251862	C	0.1	0.1	0.95	0.99 (0.80–1.22)	0.98	Shi et al.^16^
8	6	*Intergenic*	rs3800307	27293771	A	0.1	0.14	0.31	0.90 (0.75–1.08)	0.46	Glessner et al.^12^
9	6	*Intergenic*	rs6932590	27356910	C	0.2	0.2	0.07	0.85 (0.73–1.00)	0.17	Stefansson et al.^17^
10	6	*Intergenic*	rs3800318	27371620	T	0.1	0.15	0.2	0.88 (0.74–1.06)	0.37	Glessner et al.^12^
11	6	*HLA-N*	rs3130375	30429711	A	0.02	0.01	0.33	1.30 (0.77–2.20)	NA	Int Consortium,^15^
12	6	*HCP5*	rs2244839	31546347	A	0.3	0.27	0.85	1.01 (0.88–1.17)	0.55	Shirts et al.^22^
13	6	*HCP5*	rs14597	31547993	A	0.4	0.4	0.57	0.96 (0.85–1.09)	0.65	Unpublished work; dbSNP
14	6	*HCP5*	rs6940467	31550116	G	0.04	0.03	0.19	1.26 (0.89–1.76)	0.44	Unpublished work; dbSNP
15	6	*HCP5*	rs2523651	31556133	T	0.4	0.37	0.6	0.96 (0.85–1.10)	0.65	Shirts et al.^22^
16	6	*MICB*	rs6916394**	31572029	C	0.4	0.4	0.96	1.0 (0.88–1.14)	**0.02**	Unpublished work; dbSNP
17	6	*MICB*	rs3828917	31573896	T	0.04	0.05	0.42	0.86 (0.64–1.15)	NA	Unpublished work; dbSNP
18	6	*MICB*	rs3130615^##^	31583392	C	0.1	0.16	0.08	0.84 (0.71–1.01)	0.06	Stefansson et al.^17^
19	6	*PPIAP9*	rs2516489	31596017	A	0.3	0.3	0.58	0.96 (0.84–1.10)	0.86	Unpublished work; dbSNP
20	6	*NFKBIL1*	rs6916921	31628405	T	0.2	0.13	0.08	1.19 (1.00–1.43)	0.2	Unpublished work; dbSNP
21	6	*NFKBIL1*	rs2239707	31633298	G	0.3	0.31	0.59	0.96 (0.84–1.10)	0.85	Unpublished work; dbSNP
22	6	*NFKBIL1*	rs2230365	31633427	T	0.2	0.25	0.66	0.97 (0.84–1.12)	0.87	Unpublished work; dbSNP
23	6	*LTA*	rs1800610	31651806	T	0.1	0.13	0.33	1.11 (0.92–1.33)	0.56	Unpublished work; dbSNP
24	6	*LST1*	rs986475	31664688	C	0.2	0.13	0.13	1.17 (0.98–1.40)	0.32	Unpublished work; dbSNP
25	6	*NOTCH4*	rs2071278**	32273422	C	0.1	0.13	0.68	1.04 (0.86–1.25)	0.58	Stefansson et al.^17^
26	6	*Intergenic*	rs377763	32307122	T	0.3	0.27	0.58	1.04 (0.90–1.20)	0.67	Unpublished work; dbSNP
27	6	*HLA-DQA1*	rs9273012**	32719619	G	0.3	0.25	0.06	1.15 (1.0–1.32)	0.12	Glessner et al.^12^
28	6	*AHI1*	rs2064430	135684449	T	0.5	0.45	**0.04**	**1.14** (**1.0**–**1.30)**	0.13	Doi et al.^43^
29	6	*Intergenic*	rs1475069	136097927	C	0.4	0.41	0.42	1.05 (0.93–1.20)	0.66	Ingason et al.^47^
30	7	*RELN*	rs7341475	103764368	A	0.1	0.12	0.67	0.18 (0.79–1.16)	0.63	Shifman et al.^59^
31	11	*NRGN*	rs12807809	124736389	C	0.1	0.1	0.53	0.39 (0.76–1.15)	0.82	Stefansson et al.^17^
32	18	*TCF4*	rs9960767	55487771	C	0.2	0.22	0.36	0.85 (0.92–1.25)	0.51	Stefansson et al.^17^

*CA* cases, *CO* controls, *MA* Minor Allele, *MAF* Minor Allele frequency

*not in HWE; ^#^All except dbSNP, Bamne et al.^23^, Ingason et al.^47^, and Shirts et al.^22^ are GWAS reports (not from PGC-GWAS)

**rs9393709 is surrogate for rs3734536; rs6916394 is surrogate for rs3828914; rs2071278 is surrogate for rs3131296 and rs9273012 is surrogate for rs9272219

NA: one of the genotype count was less than 5, therefore excluded from analysis

^##^rs17481507 in Stefansson et al.^17^ is now merged with rs3130615 in NCBI

### Tests of association

#### Genotypic

Genotypic association was observed for SNPs rs6916394 T > C (*p* = 0.02; df = 2) and rs3130615 T > C (*p* = 0.06; df = 2) both from *MICB* (Table 3) and none of them withstood multiple testing.

#### Allelic

Modest allelic association of only one SNP namely rs2064430, *AHI1* (*p* = 0.04); and a trend for four SNPs namely rs6932590, intergenic (*p* = 0.07); rs3130615, *MICB* (*p* = 0.08); rs6916921, *NFKBIL1* (*p* = 0.08) and rs9273012, *HLA-DQA1* (*p* = 0.06) was observed with SZ. Detailed results for all the SNPs tested are presented in Table 3 and notably, none of them withstood multiple testing.

#### Haplotypic

Nominal haplotypic association (*p* = 0.01–0.05) was observed for haplotypes generated in PLINK using 1–6 marker sliding window (Supplementary Table 2) but did not withstand Bonferroni correction. SNPs identified to be associated in the univariate analysis were seen to be the drivers of associated haplotypes.

#### Association of SNPs with cognition

When the cases were compared with the controls, there was significant association of SZ status with the domains of abstraction (*p* = 1.32 × 10^–11^); attention (*p* = 0.00002); face memory (*p* = 0.00009); spatial memory (*p* = 3.14 × 10^−6^); working memory (*p* = 3.98 × 10^−7^); sensorimotor (*p* = 0.0002) and emotional processing (*p* = 2.04 × 10^−6^) (Supplementary Table 3). Six SNPs were associated with five cognition domains: *MICB*, rs6916394 with abstraction (0.04); *AHI1*, rs2064430 and *NFKBIL1*, rs2230365 (*p* = 0.05; *p* = 0.04, respectively) with face memory; rs3130375 with working memory (*p* = 0.007); rs377763 (*p* = 0.004) with sensorimotor; *NFKBIL1*, rs6916921 (*p* = 0.01) with emotion (Table 4).

**Table 4:** Association of chromosome 6 markers with cognition in the study cohort

Cognition domain (dependent variable)	Markers (gene/locus) (independent variables)	*B* value	*p*-value	95% CI
Abstraction	rs6916394 (*MICB*)	−0.12	**0.04**	**(–)0.33 to (–)0.005**
Face memory	rs2064430 (*AHI1*)	−0.11	**0.05**	**(–)0.32 to (–)0.0002**
	rs2230365 (*NFKBIL1*)	+0.12	**0.04**	**0.012–0.41**
Working memory	rs3130375 (Intergenic)	+0.16	**0.007**	**0.27–1.7**
Sensorimotor	rs377763 (Intergenic)	−0.17	**0.004**	**(–)0.47 to (–)0.09**
Emotion	rs6916921 (*NFKBIL1*)	+0.15	**0.01**	**0.07–0.57**

Supplementary Table 1

All genetic and cognitive data used in this study can be accessed at https://figshare.com/s/34a8ffa9182221b075c5.

#### Modifiers

Epistatic interactions were evaluated for all these SNPs using PLINK. We analyzed several SNPs in close proximity in the chromosome 6p21–24 region. SNP–SNP interactions were observed between rs3828917 (*MICB*) **×** rs377763 (Intergenic), rs2071278 (*NOTCH4*) **×** rs6916394 (Intergenic), rs2071278 (*NOTCH4*) **×** rs2523651 (upstream of *MICB*), and rs2244839 (*HCG26*) **×** rs1800610 (*TNF*). None of the *p-*values withstood Bonferroni corrections (Table 5).

**Table 5:** Shows significant epistatic interactions between chromosome 6 markers

Gene (SNP1)	Gene (SNP2)	Odds ratio for interaction	*χ* ^2^(1df)	Asymptotic *p*-value
*HCG26* (rs2244839)	*TNF* (rs1800610)	1.57	7.00	**0.008**
upstream of *MICB* (rs2523651)	*NOTCH4* (rs2071278)	1.55	7.81	**0.005**
*MICB* (rs6916394)	*NOTCH4* (rs2071278)	1.47	6.71	**0.01**
*MICB* (rs3828917)	Intergenic (rs377763)	0.37	9.44	**0.002**

Note: *p-*values ≤ 0.01 only included

## Discussion

Prior studies indicate that Indians have a complex population history, with similarities and differences from populations with Caucasian ancestry. Thus, studies in Indian samples might indicate susceptibility loci for SZ that are identical with, or distinct from the prior GWASs that were conducted extensively in Caucasian ancestry samples. The present study is an initial effort to extend the GWAS analyses on Caucasian populations to Indian samples. We focused primarily on the chromosome 6p region, which harbors the strongest and most consistently replicated susceptibility loci in the GWASs.^6, 16, 17, 43^ Our analyses modestly support some of the prior associations,^42, 43^ while indicating that there are possibly other associations specific to this population, which remains to be identified.

### Allelic/genotypic/hapoltypic associations

Of the 32 most significant GWAS and other prioritized SNPs, largely from chromosome 6, modest allelic/genotypic association of six SNPs namely rs6932590 (intergenic, located close to another SZ GWAS SNP rs13194053), rs6916921 (*NFKBIL1*), rs6916394 and rs3130615 (*MICB*) and rs9273012 (*HLA-DQA1*) on 6p and rs2064430 (*AHI1*) on 6q was noted (Table 2). The failure to have strong significance and to replicate other SNPs may reflect insufficient power of the study sample or differences in the genetic architecture of SZ across populations. As for the other five SNPs, though associations were only nominally significant, the findings are notable, because *NFKBIL1 and MICB* are immune response genes and previously implicated in SZ etiology.^16, 17, 44^ On the other hand, *AHI1* (located on chromosome 6q, has been reported to be associated with SZ in an Israeli Arab population^20, 45^; in Palestinian Arab families^46^ and in a meta-analysis of European populations.^47^ More recently, SNPs from this gene have been included in a SZ database (www.szgenes.org).^42, 43^ However, this gene has not been found to be associated with SZ in any GWASs or PGC GWAS. In an ab initio analysis, *AHI1* was identified as a common helper provirus integration site for murine leukemias and lymphomas.^48^ *AHI1* is a component of a protein complex in the basal body present at the base of cilia. This complex serves as a barrier between plasma and ciliary membranes to selectively restrict protein diffusion. Disruption of this complex has been shown to cause various neurological developmental abnormalities, and neurological defects are widely observed in various ciliopathies.^49^ In fact, AHI1 or Jouberin could be important for both cerebellar and cortical development in humans and mutations in this gene have been associated with Joubert syndrome, a brain developmental disorder. Multiple studies indicate that primary cilium is a sensory organelle which gathers extracellular cues that regulate brain development^50^ and may play a critical role in brain patterning.^51^

Further, epistatic interactions tested among all these functionally similar genes such as, *MICB*, *NOTCH4*,* HLA-DQA1*, *HCG26* etc. on chromosome 6, revealed a few modest associations (Table 5). Some nominally significant associations with cognitive domains were noted (Table 3). Initially, impaired cognitive performance was observed among patients with SZ for all cognitive domains except for spatial ability. Further analyses indicated that rs377763 is significantly associated with accuracy in the sensorimotor domain. This SNP is localized between *NOTCH4* and *BTNL*, both of which have been implicated in SZ susceptibility.^52, 53^ An interaction of *NOTCH4* with cognition and SZ was also reported in another study.^54^ rs3130375, the most significant SNP in an international schizophrenia consortium (ISC) study (comprised of multiple European GWASs) is associated with accuracy in the working memory domain and is localized to *RPP21*, which is involved in processing of 5′ leader sequences of precursor t-RNA. Variations in this gene are associated with monocyticleukemias.^7, 24^ SNPs in *AHI1* and *NFKBIL1* are significantly associated with accuracy in the face memory domain. Since these two SNPs were associated with variation in the same cognition domain, i.e., face memory, epistatic interactions were evaluated, revealing a modest association (*p* = 0.03).

It is now known that neurons have primary cilium which may control cerebellar morphogenesis^55^ and defects in these sensory neuronal cilia may cause some neurological diseases, as primary cilia are critical in early brain patterning and formation of adult neural stem cells.^50^ The ciliary protein complex and innate immune response genes might be interacting to keep away the unsolicited entry of pathogens. Variation in the genes governing these functions might render the person susceptible to pathogens, which may in turn disrupt the internal homeostasis and alter synaptic plasticity, thus leading to cognitive deficits.^50, 55^ Alternately, since *AHI1* is known to have a site for provirus integration and is involved in ciliary machinery that allows differential protein diffusion, and *NFKBIL1* is involved in immune response, these genes may be interacting to maintain internal homeostasis by restricting unsolicited pathogen entry. Separately, modest associations were observed with rs6916394 (abstraction domain, *p* = 0.04, *MICB*) and rs6916921 (emotion domain, *p* = 0.01,* NFKBIL1*) but how these SNPs/genes affect these cognitive domains and the role of these genes in cognition is not clear at present. A susceptibility locus conferring vulnerability to SZ with selective impairments on sustained attention on 6p for haplotype rs1225934-rs13878 in *BMP6-TXNDC5* has been reported.^56^

Some limitations of the present analyses are notable. The primary limitation is the absence of genome-wide association data in this or other Indian samples. Based on the present analyses, future GWAS in Indian samples could well identify novel genetic risk factors for SZ, addressing the missing heritability issue. Though it could be argued that some of the analyses represented tests of the prior GWAS results, the novel associations with cognition reported here need to be evaluated further in independently ascertained samples.

In summary, the present study in a north Indian SZ cohort shows very limited replication of predominantly Caucasian population-based associations reported in literature and this may be attributed to the differences in genetic architecture between the two populations or a lack of a large enough sample size. Resequencing the gene dense chromosome 6 region may facilitate identification of the primary risk variants across ethnically different populations and thereby provide new insights into the elusive SZ biology. Our study only weakly supports the prevailing notion that chromosome 6 SNPs and immune genes are involved in cognition decline and people with predilection to viral infections have a higher chance of cognitive decline and are at greater risk for developing SZ, but the probable mechanism underlying this is unclear.

## Materials and methods

### Subjects

The study was performed with Institutional ethical committee approval obtained at the participating centers i.e., Post Graduate Institute of Medical Education and Research—Dr. Ram Manohar Lohia (PGIMER—Dr. RML) Hospital and University of Delhi South Campus, New Delhi. The study was conducted in accordance with all relevant protocols. Initially, Schizophrenia cases (*n* = 1009) and controls (*n* = 1029) were recruited at PGIMER—Dr. RML hospital. Controls included age and gender balanced adult individuals without a history of psychotic illness (*n* = 507) and cord blood samples (*n* = 482). All participants completed the Diagnostic Interview for Genetic Studies (DIGS) and were evaluated according to the DSM IV criteria.^38, 40^ Written informed consent was obtained from all participants. Maternal consent was obtained for the cord blood samples. All the subjects were of north Indian origin and are genetically distinct as evidenced by admixture analysis.^26^

### Genetic analysis

Venous blood was obtained from all participants and DNA was extracted using routine phenol chloroform method.

A total of 32 SNPs (Table 3 and Supplementary Table 1) were analyzed. SNPs were selected based on available published and unpublished association studies, also keeping in view the estimated level of polymorphism and available linkage disequilibrium (LD) structure among Indians. Of these, 26 SNPs were from chromosome 6 (Supplementary Table 1). The remaining SNPs (*ZNF804A:* rs1344706 and rs7597593, *CTXN3-SLC12A2:* rs245201, *RELN:* rs7341475, *NRGN*: rs12807809, *TCF4*: rs9960767 were localized to chromosomes 2, 5, 7, 11, and 18, respectively. Of the 26 SNPs (Supplementary Fig. 1), 10 SNPs (*p* ≤ 10^−8^) were drawn from the Caucasian SZ GWAS^13, 16, 17, 57–60^ and 16 SNPs were from other published and unpublished association studies in Caucasian and African–American populations.^23^ Of note, chromosome 6p has been the most consistently associated region not only in the Caucasian SZ GWASs but also with cognition^61^ and immune dysfunction.^62^ Since our study focused on SZ and cognition, these SNPs were selected. All these SNPs had minor allele frequencies (MAF) ≥ 0.01 in the Indian samples. SNP assays were performed using Sequenom mass array according to manufacturer’s protocol, at a commercial facility. Genotypes were called using MassARRAY TYPER 4.0 genotyping software. All questionable calls were rechecked and called manually. Multidimensional scaling using PLINK indicated that the cases and controls were similar with regard to population structure (Supplementary Fig. 2).

### Cognitive data

Cognitive functions were assessed using the Hindi version of the Penn Computerized Neuropsychological Battery (CNB;^63^) as described elsewhere.^64^ Accuracy index of eight domains of cognition namely abstraction and mental flexibility, attention, facial memory, spatial memory, working memory, spatial ability, sensorimotor, and emotional equity were assessed in this study.

### Statistical analysis

Hardy–Weinberg equilibrium (HWE) checks and LD analysis was conducted using Haploview software^65^ (http://www.broadinstitute.org/scientific-community/science/programs/medical-and-populationgenetics/haploview/haploview) and PLINK(http://pngu.mgh.harvard.edu/~purcell/plink/). Allelic, genotypic, and haplotypic associations were analyzed using PLINK. Allele and genotype frequencies of each SNP were compared between patients and control groups. The minor allele was taken as a reference for allelic association (df 1); and the three genotypes formed by the two alleles were considered for genotypic associations (df 2). Pearson’s chi-square tests and odds ratios (OR) estimations at 95% confidence intervals (CI) were performed. For haplotypic association, a sliding window of 2 to 6 SNP combinations based on their consecutive physical location along a chromosome or chromosomal segment was used with default parameters. Power of the SNPs in the study was calculated using log additive inheritance model in QUANTO software (http://hydra.usc.edu/gxe/). The sample has 80% power to detect associations with SZ risk having an OR ≥ 1.4, for SNPs having minor allele frequencies (MAF) > 20%, with a disease prevalence of 1%.

Cases and controls were initially compared for demographic variables and cognitive functions using univariate analyses. Subsequently, linear regression analyses for individual cognitive domains were conducted to assess association between SNPs, cognitive domains, and health status using SPSS version 21. Since age has a significant association with cognitive function, the cognitive domains were adjusted for age.

### Data availability

All genetic and cognitive data used in this study can be accessed at https://figshare.com/s/34a8ffa9182221b075c5.

## Electronic supplementary material

SUPPLEMENTARY TABLE 1

SUPPLEMENTARY TABLE 2

SUPPLEMENTARY TABLE 3

SUPPLEMENTARY FIGURE 1

SUPPLEMENTARY FIGURE 2
