# Supplementary material for: Attempts to replicate genetic associations with schizophrenia in a cohort from north India
Source: NPJ Schizophr. 2017 Aug 30;3:28. doi: 10.1038/s41537-017-0030-8 (PMC5577284; doi:10.1038/s41537-017-0030-8)
Supplement: Supplementary file 1 — SUPPLEMENTARY TABLE 1 [file 41537_2017_30_MOESM1_ESM.docx]

**SUPPLEMENTARY TABLE 1- MAF of 32 SNPs across populations (collated from 1000 genome phase 3 database)**

|  |  |  |  |  |  |  |  | **Minor Allele frequency** | | | | | | |  |  |
| --- | --- | --- | --- | --- | --- | --- | --- | --- | --- | --- | --- | --- | --- | --- | --- | --- |
| **Sl No** | **CHR** | **Gene** | **SNP** | **BP** | **Genotype count in Affected** | **Genotype count in Unaffected** | **MA** | **Affect-ed** | **Unaff-ected** | **AFR** | **AMR** | **EAS** | **EUR** | **SAS** | **HWE** | **Source for SNP selection ^#^; reported p value** |
| 1 | 2 | ZNF804A | rs7597593* | 184668853 | 234/396/277 | 219/388/272 | C | 0.48 | 0.47 |  |  |  |  |  | **0**.**0005*** | Riley et al., 2010^60^; 0.0013 |
| 2 | 2 | *ZNF804A* | rs1344706 | 184913701 | 136/442/401 | 123/448/413 | G | 0.36 | 0.35 | 0.04 | 0.33 | 0.52 | 0.38 | 0.35 | 0.5 | O’Donovan et al., 2008^57^; 1.61 x10^-7^ |
| 3 | 5 | *CTXN3-SLC12A2* | rs245201* | 127833520 | 215/444/263 | 208/395/328 | G | 0.47 | 0.44 |  |  |  |  |  | **0.0004*** | Potkin et al., 2009^58^; 9.3x 10^-8^ |
| 4 | 6 | *BTN3A2* | rs9393709** | 26473126 | 112/431/414 | 109/464/403 | T | 0.34 | 0.35 | 0.43 | 0.30 | 0.22 | 0.40 | 0.33 | 0.18 | Int Consortium, 2009^15^; 1.18 x 10^-5^  Bamne et al., 2012^23^; 0.01 |
| 5 | 6 | *Intergenic* | rs926300 | 27167422 | 12/156/775 | 12/153/779 | A | 0.10 | 0.09 | 0.13 | 0.10 | 0.01 | 0.14 | 0.08 | 0.22 | Shi et al., 2009^16^ ; *1.1x 10^-8^* |
| 6 | 6 | *Intergenic* | rs13219181 | 27244204 | 15/171/786 | 13/168/796 | G | 0.10 | 0.10 | 0.14 | 0.11 | 0.01 | 0.14 | 0.08 | 0.31 | Glessner et al., 2009^12^; 1.29 x 10^-8^ |
| 7 | 6 | *Intergenic* | rs13194053 | 27251862 | 11/174/796 | 12/174/799 | C | 0.10 | 0.10 | 0.14 | 0.11 | 0.01 | 0.14 | 0.08 | 0.57 | Shi et al., 2009^16^; 1 x 10^-8^ |
| 8 | 6 | *Intergenic* | rs3800307 | 27293771 | 15/214/751 | 22/222/739 | A | 0.12 | 0.14 | 0.14 | 0.13 | 0.01 | 0.17 | 0.10 | 0.37 | Glessner et al., 2009^12^; 4.35 x 10^-8^ |
| 9 | 6 | *Intergenic* | rs6932590 | 27356910 | 31/291/660 | 43/313/629 | C | 0.18 | 0.20 | 0.38 | 0.20 | 0.02 | 0.24 | 0.15 | 0.74 | Stefansson et al., 2009^17^; 7.13 x 10^-8^ |
| 10 | 6 | *Intergenic* | rs3800318 | 27371620 | 20/226/736 | 21/253/711 | T | 0.14 | 0.15 | 0.25 | 0.15 | 0.03 | 0.17 | 0.11 | 0.85 | Glessner et al., 2009^12^; 6.38 x 10^-8^ |
| 11 | 6 | *HLA-N* | rs3130375 | 30429711 | 0/32/944 | 1/23/960 | A | 0.02 | 0.01 | 0.13 | 0.04 | 0.01 | 0.10 | 0.01 | 0.28 | Int Consortium, 2009^15^; 0.086 |
| 12 | 6 | *HCP5* | rs2244839 | 31546347 | 78/349/505 | 86/328/518 | A | 0.27 | 0.27 | 0.46 | 0.28 | 0.31 | 0.27 | 0.23 | 0.003 | Shirts et al., 2007^22^; 0.036 |
| 13 | 6 | *HCP5* | rs14597 | 31547993 | 146/475/361 | 161/464/359 | A | 0.39 | 0.40 | 0.29 | 0.51 | 0.55 | 0.38 | 0.47 | 0.73 | Unpublished work;dbSNP |
| 14 | 6 | *HCP5* | rs6940467 | 31550116 | 6/66/910 | 5/53/927 | G | 0.04 | 0.03 | 0.28 | 0.14 | 0.03 | 0.12 | 0.06 | 0.01 | Unpublished work;dbSNP |
| 15 | 6 | *HCP5* | rs2523651 | 31556133 | 125/462/394 | 139/452/393 | T | 0.36 | 0.37 | 0.52 | 0.71 | 0.76 | 0.66 | 0.65 | 0.60 | Shirts et al., 2007^22^; p=0.001 |
| 16 | 6 | *MICB* | rs6916394** | 31572029 | 130/504/317 | 160/444/345 | C | 0.40 | 0.40 | 0.32 | 0.40 | 0.46 | 0.32 | 0.40 | 0.44 | Unpublished work;dbSNP |
| 17 | 6 | *MICB* | rs3828917 | 31573896 | 1/87/894 | 6/88/891 | T | 0.04 | 0.05 | 0.03 | 0.05 | 0.03 | 0.05 | 0.10 | 0.07 | Unpublished work;dbSNP |
| 18 | 6 | *MICB* | rs3130615 | 31583392 | 18/236/716 | 35/243/700 | C | 0.14 | 0.16 | 0.82 | 0.82 | 0.88 | 0.73 | 0.78 | 0.02 | Stefansson et al., 2009^17^; 8.3 x 10^-11^ |
| 19 | 6 | *PPIAP9* | rs2516489 | 31596017 | 87/402/492 | 92/408/481 | A | 0.29 | 0.30 | 0.14 | 0.16 | 0.16 | 0.16 | 0.24 | 0.66 | Unpublished work;dbSNP |
| 20 | 6 | *NFKBIL1* | rs6916921 | 31628405 | 24/252/706 | 17/228/740 | T | 0.15 | 0.13 | 0.02 | 0.18 | 0.13 | 0.09 | 0.13 | 0.99 | Unpublished work;dbSNP |
| 21 | 6 | *NFKBIL1* | rs2239707 | 31633298 | 81/428/472 | 87/434/464 | G | 0.30 | 0.31 | 0.89 | 0.78 | 0.70 | 0.71 | 0.69 | 0.38 | Unpublished work;dbSNP |
| 22 | 6 | *NFKBIL1* | rs2230365 | 31633427 | 53/367/562 | 58/370/556 | T | 0.24 | 0.25 | 0.05 | 0.12 | 0.21 | 0.14 | 0.22 | 0.80 | Unpublished work;dbSNP |
| 23 | 6 | *LTA* | rs1800610 | 31651806 | 20/232/730 | 15/222/748 | T | 0.14 | 0.13 | 0.02 | 0.18 | 0.14 | 0.09 | 0.12 | 0.86 | Unpublished work;dbSNP |
| 24 | 6 | *LST1* | rs986475 | 31664688 | 24/251/707 | 19/228/737 | C | 0.15 | 0.13 | 0.03 | 0.18 | 0.15 | 0.09 | 0.13 | 0.89 | Unpublished work;dbSNP |
| 25 | 6 | *NOTCH4* | rs2071278** | 32273422 | 22/213/746 | 26/197/761 | C | 0.13 | 0.13 | 0.10 | 0.13 | 0.21 | 0.12 | 0.16 | 0.01 | Stefansson et al., 2009^14^;  2 x 10-^10^ |
| 26 | 6 | *Intergenic* | rs377763 | 32307122 | 76/397/505 | 78/379/524 | T | 0.28 | 0.27 | 0.21 | 0.15 | 0.18 | 0.18 | 0.30 | 0.52 | Unpublished work;dbSNP |
| 27 | 6 | *HLA-DQA1* | rs9273012** | 32719619 | 84/370/527 | 78/332/574 | G | 0.27 | 0.25 | 0.30 | 0.32 | 0.22 | 0.29 | 0.16 | 0.01 | Glessner et al., 2009^12^; 6.90 × 10^-8^ |
| 28 | 6 | *AHI1* | rs2064430 | 135684449 | 235/467/277 | 210/456/316 | T | 0.48 | 0.45 | 0.45 | 0.58 | 0.62 | 0.56 | 0.53 | 0.06 | Doi et al., 2012^43^;0.038 ^$^ |
| 29 | 6 | *Intergenic* | rs1475069 | 136097927 | 171/476/333 | 166/463/353 | C | 0.42 | 0.41 | 0.06 | 0.37 | 0.45 | 0.27 | 0.42 | 0.49 | Ingason et al., 2010^47^; 2.3 × 10^−4^ |
| 30 | 7 | RELN | rs7341475 | 103764368 | 17/193/772 | 14/208/762 | A | 0.12 | 0.12 | 0.17 | 0.23 | 0.07 | 0.17 | 0.12 | 0.39 | Shifman et al., 2008^59^; 9 x 10^-7^ |
| 31 | 11 | *NRGN* | rs12807809 | 124736389 | 11/171/799 | 13/179/790 | C | 0.10 | 0.10 | 0.28 | 0.14 | 0.29 | 0.17 | 0.10 | 0.32 | Stefansson et al., 2009^17^; 2 x 10^-9^ |
| 32 | 18 | *TCF4* | rs9960767 | [55487771](https://www.ncbi.nlm.nih.gov/variation/view/?q=rs9960767&filters=source:dbsnp&assm=GCF_000001405.33) | 50/343/574 | 50/320/599 | C | 0.23 | 0.22 | 0.26 | 0.06 | 0.003 | 0.06 | 0.26 | 0.65 | Stefansson et al., 2009^17^; 4 x 10^-9^ |

*Not in Hardy Weinberg Equilibrium and therefore excluded from analysis ; ** rs9393709 is surrogate for rs3734536; rs6916394 is surrogate for rs3828914; rs2071278 is surrogate for rs3131296 and rs9273012 is surrogate for rs9272219; ^$^ d value(case–control difference of allele frequencies); MA: Minor Allele; AFR: African; AMR: American; EAS: East Asian; EUR: European; SAS: South Asian respectively from 1000 genome phase 3 database;
