# Supplementary material for: Attempts to replicate genetic associations with schizophrenia in a cohort from north India
Source: NPJ Schizophr. 2017 Aug 30;3:28. doi: 10.1038/s41537-017-0030-8 (PMC5577284; doi:10.1038/s41537-017-0030-8)
Supplement: Supplementary file 2 — SUPPLEMENTARY TABLE 2 [file 41537_2017_30_MOESM2_ESM.docx]

**SUPPLEMENTARY TABLE 2 – Shows haplotypic associations of chromosome 6 markers**

**obtained using PLINK**

| SNPSs | HAPLOTYPE | F_A | F_U | CHISQ | P (df1) |
| --- | --- | --- | --- | --- | --- |
| **2 SNP window** | | | | | |
| rs6932590-rs3800318 | TA | 0.78 | 0.75 | 4.83 | 0.03 |
| rs377763-rs9273012 | GA | 0.46 | 0.49 | 4.39 | 0.04 |
| **3 SNP window** | | | | | |
| rs3800307-rs6932590-rs3800318 | TTA | 0.79 | 0.76 | 5.32 | 0.02 |
| rs6932590-rs3800318-rs3130375 | CAC | 0.08 | 0.10 | 4.17 | 0.04 |
| rs6932590-rs3800318-rs3130375 | TAC | 0.79 | 0.76 | 5.17 | 0.02 |
| rs2071278-rs377763-rs9273012 | TGA | 0.43 | 0.46 | 4.15 | 0.04 |
| **4 SNP window** | | | | | |
| rs926300-rs13219181-rs13194053-rs3800307 | TATA | 0.03 | 0.04 | 3.80 | 0.05 |
| rs13194053-rs3800307-rs6932590-rs3800318 | TTTA | 0.79 | 0.76 | 4.93 | 0.03 |
| rs3800307-rs6932590-rs3800318-rs3130375 | TTAC | 0.79 | 0.76 | 5.62 | 0.02 |
| rs2244839-rs14597-rs6940467-rs2523651 | GAAT | 0.12 | 0.14 | 4.82 | 0.03 |
| rs2244839-rs14597-rs6940467-rs2523651 | GCGC | 0.03 | 0.02 | 3.78 | 0.05 |
| rs2244839-rs14597-rs6940467-rs2523651 | GCAC | 0.08 | 0.10 | 4.34 | 0.04 |
| rs14597-rs6940467-rs2523651-rs6916394 | CACC | 0.03 | 0.04 | 5.12 | 0.02 |
| rs986475-rs2071278-rs377763-rs9273012 | CTGG | 0.04 | 0.03 | 4.55 | 0.03 |
| rs986475-rs2071278-rs377763-rs9273012 | TTGA | 0.35 | 0.39 | 6.13 | 0.01 |
| **5 SNP window** | | | | | |
| rs13219181-rs13194053-rs3800307-rs6932590-rs3800318 | ATTTA | 0.79 | 0.76 | 4.60 | 0.03 |
| rs13194053-rs3800307-rs6932590-rs3800318-rs3130375 | TTTAC | 0.79 | 0.76 | 5.45 | 0.02 |
| rs6932590-rs3800318-rs3130375-rs2244839-rs14597 | CACGA | 0.03 | 0.05 | 3.82 | 0.05 |
| rs3130375-rs2244839-rs14597-rs6940467-rs2523651 | CGAAT | 0.12 | 0.14 | 4.64 | 0.03 |
| rs3130375-rs2244839-rs14597-rs6940467-rs2523651 | CGCGC | 0.03 | 0.02 | 3.80 | 0.05 |
| rs3130375-rs2244839-rs14597-rs6940467-rs2523651 | CGCAC | 0.08 | 0.10 | 4.11 | 0.04 |
| rs14597-rs6940467-rs2523651-rs6916394-rs3828917 | CACCG | 0.03 | 0.04 | 6.09 | 0.01 |
| rs6940467-rs2523651-rs6916394-rs3828917-rs3130615 | ACTGC | 0.05 | 0.07 | 4.89 | 0.03 |
| rs2523651-rs6916394-rs3828917-rs3130615-rs2516489 | CTGTG | 0.23 | 0.21 | 4.08 | 0.04 |
| rs2230365-rs986475-rs2071278-rs377763-rs9273012 | CCTGG | 0.05 | 0.03 | 5.90 | 0.02 |
| rs2230365-rs986475-rs2071278-rs377763-rs9273012 | CTTGA | 0.26 | 0.29 | 3.78 | 0.05 |
| **6 SNP window** | | | | | |
| rs9393709-rs926300-rs13219181-rs13194053-rs3800307-rs6932590 | CTATAC | 0.02 | 0.03 | 3.71 | 0.05 |
| rs926300-rs13219181-rs13194053-rs3800307-rs6932590-rs3800318 | TATTTA | 0.79 | 0.76 | 4.46 | **0.03** |
| rs13219181-rs13194053-rs3800307-rs6932590-rs3800318-rs3130375 | ATTTAC | 0.79 | 0.76 | 5.22 | **0.02** |
| rs3800318-rs3130375-rs2244839-rs14597-rs6940467-rs2523651 | ACGCGC | 0.03 | 0.02 | 3.88 | **0.05** |
| rs3800318-rs3130375-rs2244839-rs14597-rs6940467-rs2523651 | TCGCAC | 0.01 | 0.02 | 3.97 | **0.05** |
| rs2244839-rs14597-rs6940467-rs2523651-rs6916394-rs3828917 | GCACCG | 0.03 | 0.04 | 3.91 | **0.05** |
| rs14597-rs6940467-rs2523651-rs6916394-rs3828917-rs3130615 | CACTGC | 0.05 | 0.06 | 4.96 | **0.03** |
| rs14597-rs6940467-rs2523651-rs6916394-rs3828917-rs3130615 | CACCGT | 0.03 | 0.04 | 6.23 | **0.01** |
| rs6940467-rs2523651-rs6916394-rs3828917-rs3130615-rs2516489 | ACTGCG | 0.05 | 0.07 | 5.24 | **0.02** |
| rs6940467-rs2523651-rs6916394-rs3828917-rs3130615-rs2516489 | ACTGTG | 0.24 | 0.21 | 4.43 | **0.04** |
| rs2239707-rs2230365-rs986475-rs2071278-rs377763-rs9273012 | ACCTGG | 0.04 | 0.03 | 5.63 | **0.02** |

LD was observed between rs926300 and rs13194053 (r2= 0.96) and rs6916921 and rs986475 (r2= 0.98) but they were included for haplotype analysis
