# Supplementary material for: Attempts to replicate genetic associations with schizophrenia in a cohort from north India
Source: NPJ Schizophr. 2017 Aug 30;3:28. doi: 10.1038/s41537-017-0030-8 (PMC5577284; doi:10.1038/s41537-017-0030-8)
Supplement: Supplementary file 3 — SUPPLEMENTARY TABLE 3 [file 41537_2017_30_MOESM3_ESM.docx]

| **Cognition domain (Dependent variable)** | **B value** | **p value** | **95% CI** |
| --- | --- | --- | --- |
| Abstraction | (-)0.40 | **1.32 x 10^-11^** | (-)1.10 - (-)0.59 |
| Attention | (-)0.27 | **0.0002** | (-)0.84 - 0.27 |
| Face memory | (-)0.23 | **0.00009** | (-)0.84 - (-)0.36 |
| Spatial Memory | (-)0.28 | **3.14x10^-6^** | (-)0.75 - (-)0.33 |
| Working memory | (-)0.32 | **3.98 x 10^-7^** | (-)0.92- (-)0.41 |
| Sensorimotor | (-)0.23 | **0.0002** | (-)0.72 - (-)0.23 |
| Emotion | (-)0.29 | **2.04 x 10^-6^** | (-)0.85- (-)0.36 |

**SUPPLEMENTARY TABLE 3: Association of cognitive domains with schizophrenia**
