# Supplementary material for: Attempts to replicate genetic associations with schizophrenia in a cohort from north India
Source: NPJ Schizophr. 2017 Aug 30;3:28. doi: 10.1038/s41537-017-0030-8 (PMC5577284; doi:10.1038/s41537-017-0030-8)
Supplement: Supplementary file 4 — SUPPLEMENTARY FIGURE 1 [file 41537_2017_30_MOESM4_ESM.docx]

**SUPPLEMENTARY FIGURE 1-SNPs on chromosome 6p analysed in this study**
